# Variability of transposable elements in six genetic isolates from North-Eastern Italy and their relationship with alcohol consumption, tobacco use and BMI

**DOI:** 10.1186/s12864-025-12225-1

**Published:** 2025-11-11

**Authors:** Giorgia Modenini, Giacomo Mercuri, Paolo Abondio, Giuseppe Giovanni Nardone, Aurora Santin, Paola Tesolin, Beatrice Spedicati, Alessandro Pecori, Giulia Pianigiani, Maria Pina Concas, Giorgia Girotto, Paolo Gasparini, Alessio Boattini, Massimo Mezzavilla

**Affiliations:** 1 Department of Neuroscience “Rita Levi Montalcini”, University of Turin, Turin, Italy; 2 Department of Chemistry, Life Sciences and Environmental Sustainability, University of Parma, Parma, Italy; 3 Department of Biology, University of Rome Tor Vergata, Rome, Italy; 4 Department of Medicine, Surgery and Health Sciences, University of Trieste, Trieste, Italy; 5 Institute for Maternal and Child Health – IRCCS, “Burlo Garofolo”, Trieste, Italy; 6 BiGeA Department, University of Bologna, Bologna, Italy; 7 Department of Biology, University of Padua, Padua, Italy

**Keywords:** Genetic isolates, Polymorphic transposable elements, Constrained genes, Behavioral traits

## Abstract

**Background:**

Half of the human genome is derived from Transposable Elements (TEs), among which Alu, LINE-1 and SVA are particularly represented. Germline transposition of TEs generates polymorphisms between individuals and may be used to study association with phenotypes and inter-individual differences. Italy presents an increased number of isolated villages compared to other European groups, and these isolates provide a desirable study subject to help understanding the genetic variability of the Italian peninsula. Therefore, we focused on the relationship between polymorphic TEs, behavioral traits (tobacco use and alcohol consumption), and Body Mass Index (BMI) variations, which could lead to an increased risk of developing addiction-related or metabolic diseases.

**Results:**

We identified 12,709 polymorphic TEs in 586 individuals from six isolates: classical population genetics analyses showed that while closely related to other European populations, the isolates tend to cluster amongst themselves and are dominated by drift-induced ancestral components. Several TEs in constrained genes were also significantly related with behavioral traits (tobacco use or alcohol consumption) or with BMI variations and some of them have a functional role.

**Conclusions:**

These results suggest that polymorphic TEs may significantly impact inter-individual and inter-population phenotypic differentiation, while also functioning as variability markers and potentially having a role in susceptibility to medical conditions.

**Supplementary Information:**

The online version contains supplementary material available at 10.1186/s12864-025-12225-1.

## Introduction

The Italian peninsula, due to its complex population structure, could play an important role in the understanding of the genetic diversity of current populations, being the natural crossroad for human migrations across the Mediterranean since prehistoric periods. These migration patterns left a tangible mark on present-day Italians, revealing a heterogeneous network of genomic landscapes across the peninsula, with North Italian groups being more closely related to Western/Eastern European populations and a progressively increasing genetic connection with Northern African and Middle Eastern populations as we move southwards [1]. On top of this clinal variation across the peninsula, the natural variety of environments [2] provoked a series of local adaptive events that determined, among other factors, a differential disease susceptibility of Italian subpopulations [1]. A refined understanding of these local events would improve our knowledge of human diversity as a whole, and on a more practical level allow us to provide more ad hoc medical care and measures to particularly susceptible subpopulations.

The underlying genetic variability of Italy remains under-sampled and underrepresented, with available human genome reference datasets such as the 1KGP, HGDP, and SGDP only sampling three populations for the whole peninsula: Tuscans (TSI, 113 individuals), Bergamo (14) and Sardinians (28), a notion that only worsens when considering that Italy presents an increased number of historically isolated villages and subpopulations when compared to other European groups [3, 4], most of which remain uncharacterized.

These groups provide a desirable study subject to understand the Italian genetic variability: population isolates are characterized by small effective population sizes (Ne), which result in a decreased variability and stronger genetic drift effects, potentially increasing the frequency of variants that are rare or absent elsewhere and aiding at the discovery of novel rare variant signals underpinning complex traits such as medical risks and susceptibilities [5]. Population isolates tend not only to be genetically homogenous but are also characterized by an elevated diversity when compared to neighboring populations and their source population [3], because of geographical and/or cultural barriers that are necessary for the formation of the isolate in the first place. For these reasons, isolates can be useful tools for genome-wide association studies [6].

However, most of the available research on these populations is based almost exclusively on SNP data, while little work was done using other types of genetic markers. For instance, information about the variability of Transposable Elements (TE), despite them being a primary component of the human genome, has become accessible only in recent years, thanks to the availability of whole-genome sequencing data and in particular to the development of new tools for their detection and genotyping [7–9]. When TEs transpose in the germline, they can create novel inheritable insertions, thereby generating human-specific polymorphisms [7]. One of the most useful features of polymorphic TEs is that the ancestral state of these markers is known to be the absence of the insertion [10, 11]. Interestingly, such markers have never been used to study the genetic underpinnings of human isolated communities; therefore, this study is the first of a kind.

In the last decades, we have come to know much more about the impact of these elements on the genome and gene networks, and it has been shown that TE insertions can generate diversity in a variety of ways. For example, transposable elements have been linked to providing polyadenylation signals inducing the termination of transcripts [12], modifying splicing patterns, and providing new splicing sites [13], epigenetically affecting nearby genes [14, 15], acting as novel promoters, enhancers, and transcription factor building sites [16, 17], and often carrying their enhancers and promoters [18]. With their innate ability to act as disruptors and deregulators of gene expression, TE insertions have been associated with a variety of human diseases: for example, several cancer types [19, 20], hemophilia A and B [21, 22], some inheritable genetic diseases such as Dent’s disease or Duchenne muscular dystrophy [23], metabolic diseases [24], substance abuse, and central nervous systems diseases [25].

In particular, much interest has been given in recent years to the impact of transposable elements on the central nervous system [25–27]. Genome-wide approaches allowed researchers to study the role of transposable elements in stress-related learning mechanisms in rats [28], which have been used as a model for PTSD in humans [29]. Likewise, transposable elements have also been associated with alcoholism in humans using the same genome-wide approach [25].

In this study, we aim to reconstruct the TE variability of six isolates from Friuli-Venezia Giulia (North-Eastern Italy) thanks to the availability of whole sequencing data from 589 individuals [3]. Firstly, after determining the position and the genotypes of polymorphic TEs in these populations, we use them to evaluate the isolates’ structure, in the context of European and worldwide reference populations. Then, leveraging on the advantages offered by genetic and geographic isolates, we focused on exploring the potential association between non-reference polymorphic TEs, Body Mass Index (BMI) variations and behavioral traits of health and social relevance such as tobacco use and alcohol consumption. In fact, these traits could lead to an increased risk of developing addiction-related or metabolic diseases, such as tobacco use disorder, alcohol use disorder, and obesity [30–32].

## Methods

### Genomic and phenotypic data selection

**Fig. 1:**
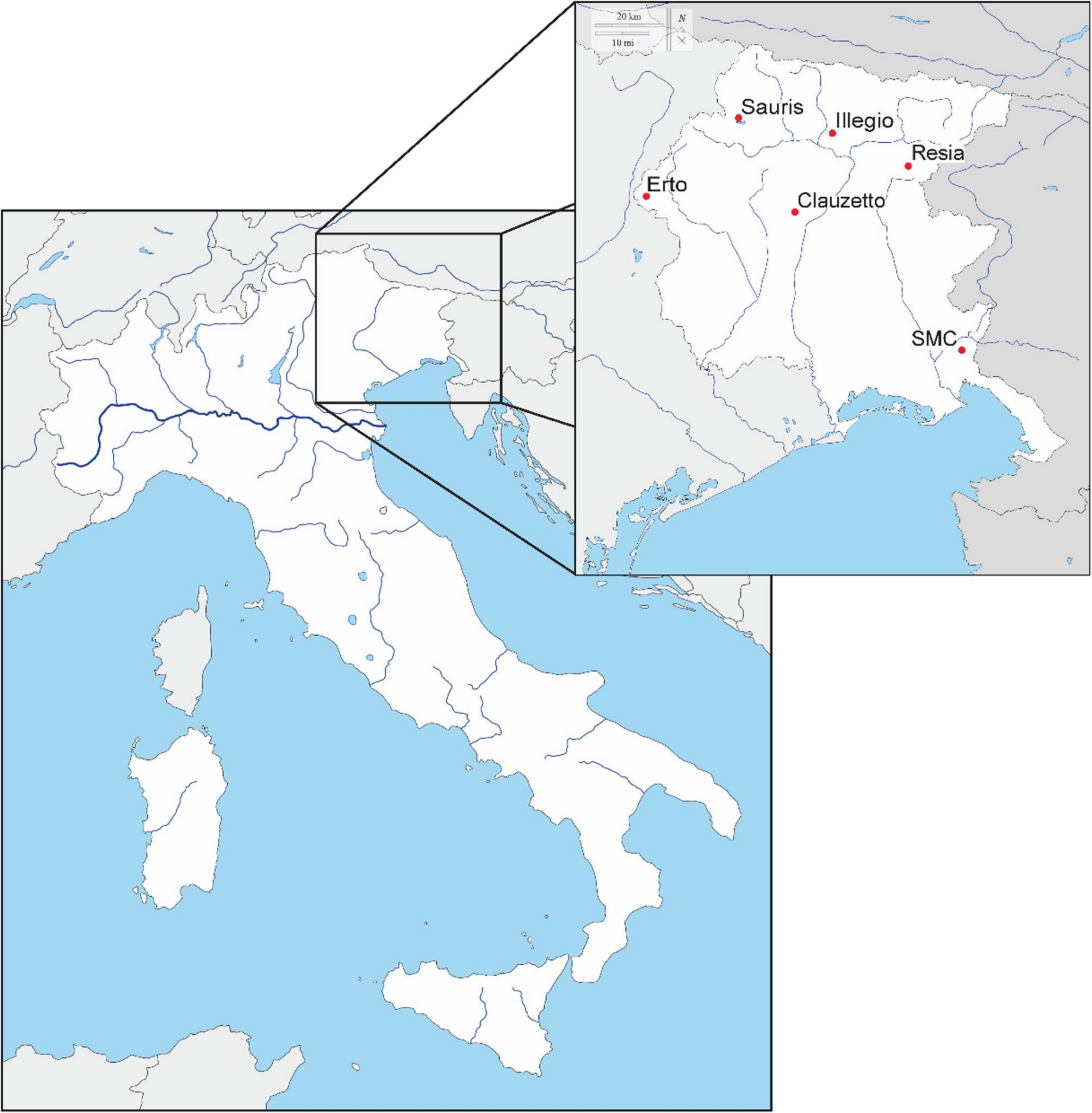
Location of the six isolates in Friuli-Venezia Giulia, north-east of Italy (SMC = San Martino del Carso)

The dataset used in this study was generated in 2008 [3–5] from the sampling of 611 individuals from six geographically and historically isolated villages in the Friuli-Venezia Giulia region of North-Eastern Italy, namely Sauris, Illegio, Resia, Erto, Clauzetto and San Martino del Carso (Fig. 1). As described in Cocca et al., 2020 [4], samples were “genotyped using the Human370CNV according to the manufacturer’s protocol (Illumina Inc., San Diego, CA, USA). The published data sets used in the analysis had been genotyped with different versions of Illumina beadchips”. Since a few of the individuals present in the dataset were duplicates (specifically, 22 individuals from Resia), and three missed village information, they were removed, leading the total number of analyzed individuals to 586. During the sampling, subjects were asked to fill out an anamnesis form to acquire more data on their general health and lifestyle habits. Phenotypic data on more than 70 traits was collected, also including food preferences, olfactory perception, gustatory perception and anthropometric measures. Since the form was administered at individual discretion, missing rates vary wildly between phenotypes and individuals. We chose to focus solely on phenotypes exhibiting a missing rate of less than 10% in our dataset, thus the traits included in our analysis were sex, age, alcohol consumption, smoking, as well as height and weight, from which we calculated the corresponding BMI (weight/height^2^). Phenotypes linked to specific diseases or health conditions, such as the occurrence of diabetes, displayed a missing rate exceeding 40%, and as such we chose to not include them in our analyses.

### Transposable elements’ variant calling and filtering

Genomes were scanned in search of non-reference polymorphic TEs (Alus, LINE1s, and SVAs), using the Mobile Element Locator Tool (MELT) v2.2.2 [8]. The WGS data was aligned with *bwa* [33] to the human reference Human GRCh38, and the aligned reads were used as input for MELT. For the calling process, we used the MELT mobile element reference sequences and the collection of insertion sites discovered in Phase III of the 1000 Genomes Project (1KGP) as analysis priors. After the identification of these TEs, a self-customized Python script was applied to the resulting *vcf* files to calculate both allele and genotype frequencies of each TE for all the isolated villages. Allele frequencies were then analyzed for significant differences between villages with Fisher’s exact test, using a significant threshold of nominal p-value < 0.01 (“differentiated” TEs).

MELT provides gene names in RefSeq format: therefore, RefSeq accession numbers were converted to their respective Official Gene Symbol using the Database for Annotation, Visualization and Integrated Discovery (DAVID) (https://david.ncifcrf.gov/*)* [34], taking into consideration the specific gene region TEs were inserted in (Intron, Exon, Promoter, Terminator, 5’ UTR and 3’ UTR).

To compare TE diversity of the isolates with other human populations, we built a new dataset consisting of polymorphic TEs identified with MELT [8] that were present both in the six isolates and in the populations of the 1000 Genomes Project. This newly merged dataset contained a total of 2,814 genetic loci for 3,090 individuals from 32 populations. The populations were divided into 6 groups based on geographic macro areas, consistent with the super populations of 1KGP [35], i.e. Africa (AFR), America (AMR), East Asia (EAS), Europe (EUR), South Asia (SAS), plus the isolates from Friuli-Venezia Giulia (FVG).

In order to perform downstream analyses, TEs were coded as single nucleotide variants. In particular, since the original MELT output codes the absence of a TE with a single base, we substituted the presence of the insertion with another nucleotide base that was non-complementary to the previous one, according to the following pattern: A/T → C; C/G → A. Information about the true nature of each insertion was kept in the original *vcf* file. Variants were then filtered with PLINK v1.9 [36] as follows: (1) Removal of insertions located on sexual chromosomes or mitochondrial genome insertions, to retain only autosomal variability and removal of duplicates, using the --*exclude* option. (2) Exclusion of individuals and variants with >1% missing data with the commands --geno 0.01 (for variants) and --mind 0.01 (for individuals). (3) Removal of variants that did not respect the Hardy-Weinberg Equilibrium (HWE) with the option --hwe, setting a significant threshold of 0.01 using a Bonferroni Correction for multiple testing (threshold = 0.01/number of variants). This was done to ensure the removal of potential genotyping errors, a common procedure in large genomic datasets [37]. (4) Removal of variants with a minor allele frequency < 0.01 (--maf 0.01). (5) Removal of closely related individuals with an Identity by Descent (IBD) estimate higher than 0.25, using the --genome option to calculate the pairwise IBD estimates between every couple of individuals and --remove to exclude one of the two related individuals. Therefore, the final filtered dataset was made of 1,703 variants shared among 3,087 individuals.

### Exploration of population structure

The generated dataset was then used to perform a series of analyses on TE insertions from the six isolates when compared to 1KGP groups. Both a Principal Component Analysis (PCA) and Admixture analysis were applied: PCA was performed after the conversion from the PLINK format (*bed*,* bim*,* fam*) with the *convertf* and *smartpca* tools of the EIGENSTRAT v6.0.1 package [38]. Admixture was implemented with the ADMIXTURE tool [39], testing between 2 and 23 potential ancestry components (K) and performing 50 iterations of each run to minimize the estimation error and maximize the *log-likelihood* of each ancestry estimate.

We then compared FVG isolates with other European populations, subsampling the original 1KGP dataset as follows: Utah residents with North-Western European ancestry (CEU), Finnish in Finland (FIN), British in England and Scotland (GBR), Iberian populations in Spain (IBS), Tuscans in Italy (TSI). PCA and Admixture analyses were implemented using the above approach, the only difference being that we tested a number K of putative ancestry components between 2 and 12. Finally, we used *TreeMix* [40] to construct a phylogenetic tree based on mobile elements’ variability and using representative populations from Europe, as well as the Yoruba as an outgroup.

### Evaluation of genes under genetic constraint using GEMMA

As introduced in the “background” section, individuals were asked to fill out an anamnesis form, including information on their health status and lifestyle habits. With the aim of exploring the relationships between polymorphic TEs and specific phenotypes or traits from our isolates, we focused on the following: tobacco use, alcohol consumption, and body mass index (the latter was calculated as weight/height^2^). Being aware that our dataset lacks the power to perform a full-scale genome-wide association study, we focused our attention on a set of constrained genes. In particular, we collected measures of genetic constraints such as pLI (probability of loss of function intolerance) and missense Z score [41], RVIS (Genic Intolerance) [42] and SSC score (Singletons Score) [43] for prioritization. We considered as constrained those genes with pLI >0.9 or Missense Z score >95th percentile of the genomic distribution or RVIS < 5th percentile of the genomic distribution or SSC score < −2, finally resulting in a total of 4067 genes analyzed.

Then, we performed a series of tests using the software GEMMA [44, 45] by applying for all the considered phenotypes a univariate linear mixed model (uvLMM) for tests between a marker, a chosen phenotype, and any chosen covariates, while also correcting for the potential presence of population stratification (indeed a typical feature of isolates), and estimating genetic correlation among phenotypes [45]. GEMMA was applied to the full FVG dataset (12,709 TEs and 586 individuals) and three separate uvLMM analyses were performed, using sex and age as covariates: (1) BMI; (2) a binary alcohol drinker/non-drinker variable (set as “1” for drinker individuals and “0” for non-drinkers); (3) a binary smoker/non-smoker variable (using “1” for smokers and “0” for non-smokers). A fourth test on the smoker individuals was performed to evaluate the possible association between polymorphic TEs and the number of cigarettes smoked per day/number of years smoking. Then we selected only the transposable elements that fulfil the following criteria: they should map inside a gene, and that gene should have at least two indicators of genetic constraints; the resulting number of Gene/Alu used for the association analyses was 105.

Then TEs were tested using Wald’s test with a significant threshold of p-value < = 0.05/105 (0.000454).

### Haplotype-based analyses

To better assess the importance of the identified TEs, we also performed a haplotype reconstruction/association test procedure on the significant variants from the alcohol and smoking tests detected with GEMMA using Beagle [46], choosing these traits as Beagle only performs association tests on binary variables. First, we selected regions of interest (10 kb upstream and downstream the significant TE, for a total of 20 kb) with VCFtools [47] and phased those regions with the software Beagle v5.1. The obtained *vcf* files were converted into the Beagle format with vcf2beagle (https://faculty.washington.edu/browning/beagle_utilities/*)* and the case status “smoking” or “alcohol” was included in the second row of the *bgl* files. Lastly, the association test on the reconstructed haplotypes was performed with Beagle v3.3.2 and the significant results were checked with the cluster2haps utility. In order to investigate a possible function for the identified TEs, we then cross-checked the significant results with the lists of polymorphic TEs acting as expression/alternative splicing quantitative trait loci produced by Cao and colleagues [48].

## Results

### TE variation distribution

After the analysis of polymorphic non-reference TEs with MELT v.2.2.2 [8], a total of 9,525 Alus, 2,283 LINE1s, and 901 SVAs were retrieved.

Then, allele frequencies were scanned for significant differences among the isolates: this way, a total of 3,987 TEs (31.37%) were identified as “differentiated”, of which 3,195 Alus (33.54%), 636 LINE1s (27.86%), and 156 SVAs (17.31%). When considering all comparison European populations, the corresponding rates of “differentiated” TEs are 53.45% (Alus), 58.63% (LINE1s) and 51.24% (SVAs).

 Of these insertions, we also considered their location (Table 1).

**Table 1:** Significantly different polymorphic TEs between the six villages, divided by insertion location relative to gene region (with percentages) and TE superclass

	Alu	LINE1	SVA
INTRONIC	1,281 (40,1%)	242 (38%)	65 (41,7%)
PROMOTER	138 (4,3%)	23 (3,6%)	10 (6,4%)
TERMINATOR	106 (3,3%)	28 (4,4%)	11 (7%)
EXON	38 (1,2%)	6 (1%)	6 (3,8%)
3’-UTR	39 (1,2%)	6 (1%)	3 (1,9%)
5’-UTR	18 (0,6%)	2 (0,3%)	0
INTERGENIC	1,575 (49,3%)	329 (51,7%)	61 (39,1%)
TOTAL	3,195	636	156

As expected, most polymorphic TE insertions are located in intronic and intergenic regions and only a negligible fraction are located in exonic regions (Supplementary Table S1). However, it is interesting to note that SVAs, which can be up to 3 kb long [49], are overall less frequent in intergenic sequences while they appear more often located in “functional” regions (regulators or exons) when compared to Alus and LINE1s. This finding corroborates the notion that SVA insertions have the innate potential to regulate gene expression through their location insertion and their sequence characteristics [50, 51].

### TE as markers for population structure

Both TE-based PCA and Admixture show that, while closely related to other European populations, our isolates tend to cluster amongst themselves and are dominated by drift-induced ancestry components (Supplementary Figure S1). In particular, the first PC discriminates between African and non-African populations, while the second PC highlights a West-to-East geographical pattern including individuals from Friuli-Venezia Giulia, Europeans, Americans, South Asians, and East Asians.

**Fig. 2:**
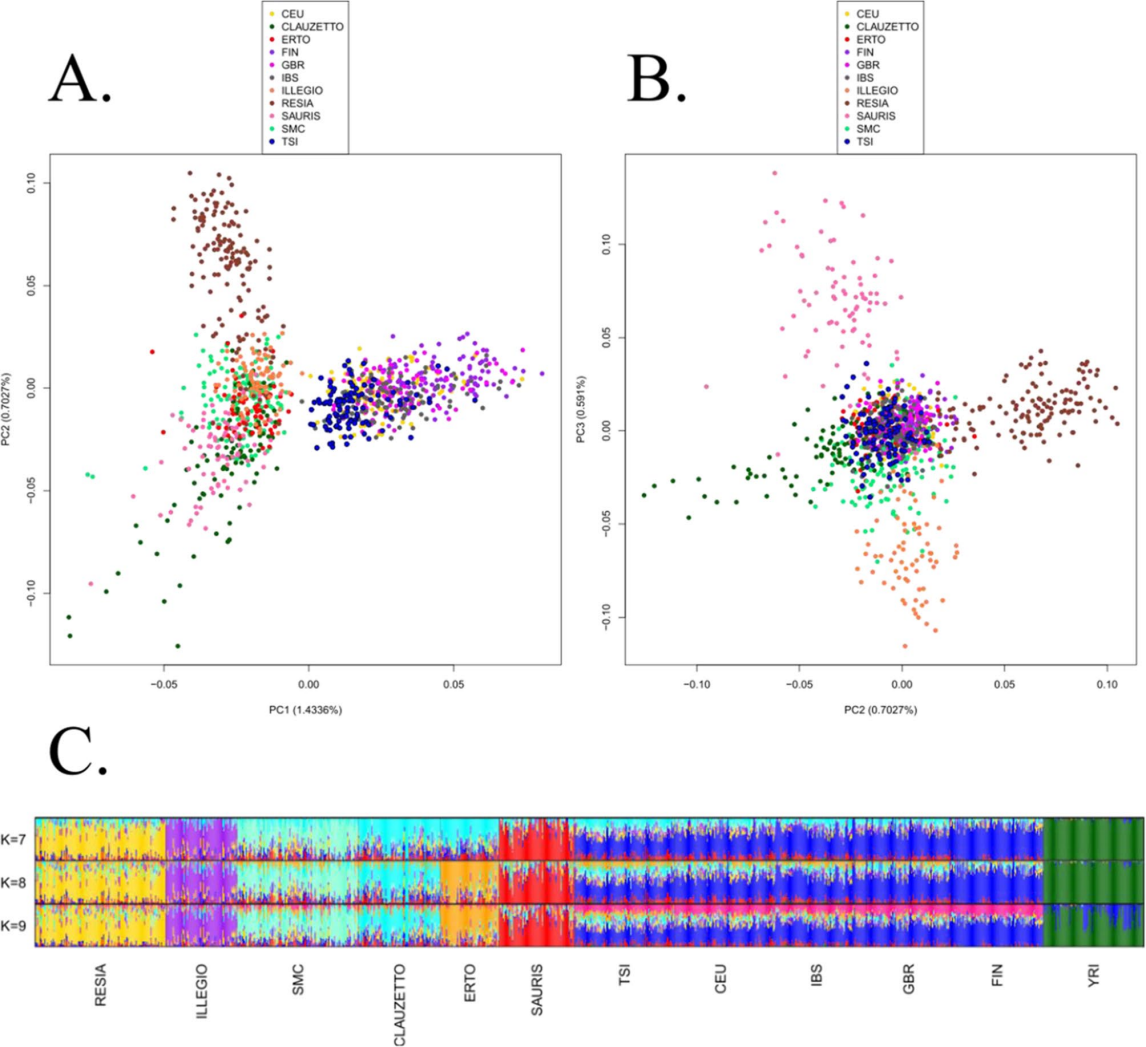
**A** and **B**) PCA plots of European populations from 1KGP and FVG isolates, first against second component (**A**) and second against third component (**B**). **C**) Admixture barplots for K = 7–9

The PCA including only European and FVG populations divides the two groups along the first PC, while the second component highlights the variability between the isolates, separating Resia and some individuals from Clauzetto and Sauris from the rest (Fig. 2A). As expected considering their geographical proximity and historical relatedness, Tuscans (TSI) and Central Europeans (CEU) are the closest groups to the FVG isolates. This PCA is similar to the one resulting in Esko et al. [3] based on SNPs. Looking at the second and third PCs, it is interesting to note that PC2 separates Resia from Clauzetto, while the third component highlights the differentiation between Sauris and Illegio. Instead, Erto, San Martino and most individuals from Clauzetto cluster together with the other European populations (Fig. 2B), hence suggesting a lower degree of isolation for these groups. Finally, looking at the Admixture graph (Fig. 2C; Supplementary Figure S2), models start “tidying” up at K = 7, with Erto and Clauzetto sharing their dominating ancestry, and reach the most supported model at K = 9 (Fig. 2C; Supplementary Figure S2; Supplementary Table S2). However, the “tidiest” model is for K = 8, as results for K = 9 and onward present excessive noise (Supplementary Figure S2), adding further ancestry components to European populations and to the African outgroup, which are unnecessary or even confounding for FVG populations. Interestingly, at K = 8, all isolated villages are dominated by their own ancestry component; on the contrary these village-specific components are present only marginally in the other European populations. Accordingly, the phylogenetic tree obtained with *TreeMix* (Supplementary Figure S4), from one side recapitulates well known relationships among the considered populations, from the other it shows that all FVG isolates cluster together. When migration edges were taken into consideration, the tree with 5 edges (explaining 99.9% of variation) showed no signals of gene flow from European populations to the Friulan isolates (Supplementary Figure S5).

### Association between TEs in constrained genes and selected phenotypes

**Fig. 3:**
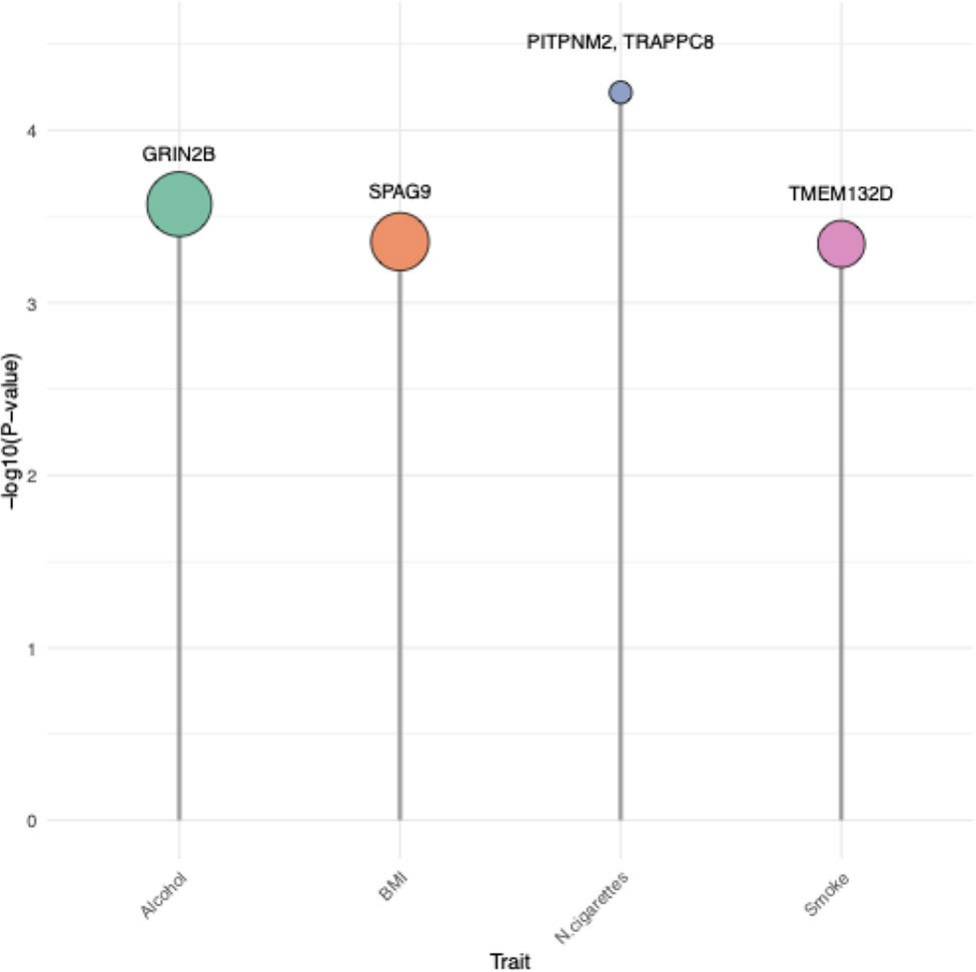
Results for association tests in constrained genes. Genes are divided by tested traits (BMI variation, alcohol consumption, smoking, and number of cigarettes smoked per day/years smoking). Balloon size is proportional to the average constraint score of genes inside of it

We focused on genes under evolutionary constraint, based on the hypothesis that transposable elements (TEs) are more likely to influence phenotypes when inserted in genes subject to purifying selection. Therefore, we analyzed only genes that showed evidence of constraint according to at least two of the following metrics: pLI [41], missense Z-score [41], RVIS [42], or SSC score [43]. We found that some polymorphic TEs (Fig. 3) are possibly associated with the conditions detailed in Materials and Methods, and some of them also act as eQTLs/sQTLs. More in detail:

1. Variations in Body Mass Index: three insertions were deemed significant, namely two Alus and one SVA). Notably, the SVA on chr17:49150166 is located in the gene SPAG9 (Sperm Associated Antigen 9), labelled as constrained by pLI, missense Z score, SSC score and RVIS (Fig. 3; Table 2).
2. Alcohol consumption: one Alu was found to be significant in a genic region, the Alu on chr12:14020945 in the gene *GRIN2B* (Glutamate Ionotropic Receptor NMDA Type Subunit 2B; Fig. 3). This TE was previously identified as “differentiated” among the isolates and is generally widespread in our six villages (Table 2).
3. Tobacco use (smoking): only the Alu on chr12:129970510 in *TMEM132D* (Transmembrane Protein 132D), labelled as constrained, was found associated (Fig. 3). This Alu is mostly widespread in the six considered villages (Table 2) and was identified as “differentiated” between the isolates (Table 2).
4. A further test with GEMMA was performed on the “smoking” condition by taking into account the number of cigarettes smoked per day and the number of years smoking.). Significant results include the Alu on chr12:123580101, located in the gene *PITPNM2*(Phosphatidylinositol Transfer Protein Membrane Associated 2); and the Alu on chr18:29519986, located in the gene *TRAPPC8 *(Trafficking Protein Particle Complex Subunit 8) (Figure 3). The full GEMMA output for each association analysis, including per trait effect size, variance, standard error, and associated *p-value* of each significant insertion, is reported in Supplementary Table S3.

Absolute genotype frequencies of these insertions are reported in Table 2. The Alus in the genes *SPAG9*,* PITPNM2*, and *TRAPPC8*, despite being significant, appear to be rare, therefore we did not report the genotype frequencies in Table 2 (the percentage of individuals who carry the insertion is 1.9% for SPAG9, 0.18% for *PITPNM2*, and 0.26% for *TRAPPC8*).

We finally reconstructed haplotypes around the above mentioned TEs and performed haplotype-based association tests as described in Methods. We obtained two significant results, both for the alcohol phenotype, namely the two intergenic Alus on chr6:1257163 and chr6:161283170. In both cases, the associated haplotype is characterized by the presence of the mobile element. The first haplotype included 19 SNPs and one Alu (p-value = 0.00164); the second is a haplotype with 7 SNPs and the TE (p-value = 0.000335).

**Table 2:** Absolute genotype frequencies of the two alus located in the genes *GRIN2B*, and *TMEM132D*

	Resia			Erto			Illegio			Sauris			San Martino			Clauzetto		
	0\0	0\1	1\1	0\0	0\1	1\1	0\0	0\1	1\1	0\0	0\1	1\1	0\0	0\1	1\1	0\0	0\1	1\1
**GRIN2B**	53	60	30	27	30	6	23	37	17	35	39	11	52	70	8	32	51	5
**TMEM132D**	75	57	11	22	30	11	42	31	4	45	38	2	46	67	17	38	38	12

## Discussion

The study of isolated communities is at the basis of population genetics research [52, 53]. In fact, isolates yield genomes that show high homogeneity and are subject to similar environmental and cultural pressures, such as lifestyle habits, diet, sanitary conditions, and disease vectors. These populations also can be of help to study the phenotypic effects of variants that were otherwise only marginally present in larger populations [53]. In this picture, Italian ​​isolates are particularly important, mainly because of the peninsula’s central role in human migrations since prehistoric times and of the high number of genetically distinct isolated communities that have been established throughout history [54]. Polymorphic TEs, which have previously been used as both variability and susceptibility markers only in “general” populations [7, 8, 25], are here applied for the first time to human isolates. Using the Mobile Element Locator Tool [8] more than 12,000 polymorphic TEs were identified in the six villages of Friuli-Venezia Giulia. These TEs were used as genetic markers to obtain a first overview of their potential impact on diversity and disease susceptibility in isolated populations, in particular: (1) to study communities’ differentiation; (2) to explore the genetic variability of the isolates; (3) and to analyze their possible role as genetic variants underlying susceptibility to different behavioral traits or medical conditions (tobacco use, alcohol consumption, and BMI variations).

Firstly, after calculating allele and genotype frequencies of the identified TEs, we found that of 12,709 TEs, 3,987 (31.37%) have significantly different allele frequencies between the six isolates (Fisher’s exact test, p-value < 0.01), while the corresponding rate in European comparison populations is 53.78%. Considering the much lower geographic dimensions of FVG compared to Europe, these values suggest the presence of genomic structure among the isolates.

Then, TEs were used as markers for exploratory population analyses, such as PCA, Admixture and TreeMix, to look at the general diversity and ancestry of FVG isolates in the context of European genetic variability, as represented by the polymorphic TE content of European populations from 1KGP [8]. Our results show that FVG isolates tend to cluster amongst themselves (PC1 in Fig. 2A and C, phylogenetic tree in Supplementary Figure S4), compared to European populations; however some differentiation between the isolates is evident, particularly for Resia and some individuals from Clauzetto (PC2 in Fig. 2B and C), as well as Sauris and Illegio (PC3 in Fig. 2B and C). Instead, Erto, San Martino and most individuals from Clauzetto overlap with the other European populations. These results agree with previous SNP-based studies, according to which, Clauzetto is the least isolated village among the six FVG isolates [3]; at the same time Clauzetto, Erto and San Martino have the lowest inbreeding coefficients among the villages [4]. The observed patterns of genetic variability and ancestry components could be explained by population structuring and genetic drift, a suggestion made also by previous works on the same dataset [3–5]. The observation of a strong correlation between SNP-driven results and TE-driven results in terms of population structure further highlights that the variability of polymorphic TE is mainly the result of demographic events.

To sum up, population structure analyses confirmed that on the whole, our populations show the typical marks of isolates also from the TEs point of view. As previously mentioned, due to their internal homogeneity both at genetic and social levels, they may be of help to perform genome-wide association studies. On the other hand, their relatively low census size implies a moderate number of available samples [and makes highly unlikely the availability of replication cohorts]. More importantly, the presence of population structure is well known to induce false positives in association studies. However, the impact of the observed structure is probably moderate or at least not higher than in association studies at a country level, as suggested by exploratory population analyses (PCA, Admixture) and proportions of “differentiated” TEs. In addition, the usage of GEMMA should overcome distortions due to population structure, as confirmed by the fact that only a minority (3/22) of the associated variants show significant differentiation among isolates.

In this context, polymorphic TE insertions are particularly worthy of investigation, being potential risk variants for several medically relevant phenotypes, because of their innate ability to act as deregulators of gene networks [15]. Notably, the link between transposable elements and the health of the Central Nervous System is not new [26, 27], with the effects of TEs being associated with stress, neurodegeneration, ageing, and drug abuse [25]. As such, TE markers can allow us to perform a first exploration of the medical susceptibility of individuals from the studied villages, by testing for association between TEs and phenotypes linked to behavioral and anthropometric traits.

Since the characteristics of our isolates (small sample size and lack of a replication cohort) do not allow to directly perform a “classic” genome-wide association study, we adopted a prioritization approach using only genes under putative purifying selection, followed by an association-like analysis to perform an initial exploration of this potential connection. Accordingly, we used GEMMA [44, 45] in order to obtain a first overview of the polymorphic TEs in constrained genes that could underpin the variability of selected phenotypes, i.e. tobacco use, alcohol consumption, height and weight, from which we calculated body mass index (weight/height^2^). For tobacco use, two separate analyses were run, the first comparing smokers with non-smokers, the second only on smoker individuals, testing for the association between the number of cigarettes smoked per day and the number of years smoking. In addition, sex and age were introduced in the models as covariates. Several TEs were deemed significant, some of which are located in known genes, as shown in Fig. 3: an SVA (chr17:49150166) in the gene *SPAG9* (BMI variations); the Alu on chr12:129970510 in *TMEM132D* (tobacco use/smoking) and the Alu on chr12:14020945 in the gene *GRIN2B* (alcohol consumption). Interestingly, the alcohol consumption phenotype was deemed significant also for the haplotype-based association test performed with Beagle [46], and the haplotype including the polymorphic TEs appears as significantly linked with the status “alcohol drinker”. As for the amount of cigarettes/number of years smoking, it resulted as of interest two Alu insertions: one on chr12:123580101, in the *PITPNM2* gene, and one on chr18:29519986, in the *TRAPPC8* gene. Additionally, the insertions in *TMEM132D* and *GRIN2B* were also identified as “differentiated” when looking at genotype and allele frequencies between the isolates. For instance, the gene *GRIN2B* encodes a member of the ionotropic glutamate receptor superfamily and plays a major role in brain development and synaptic plasticity, with mutations in this gene often associated with neurodevelopmental disorders [55]. In addition, variants of this gene have been associated with alcohol and tobacco consumption [56], general risk-taking behaviors [57], opioid dependence [58], and several neurological disorders such as schizophrenia [59] and Alzheimer’s disease [60]. Furthermore, *TMEM132D*, encoding for a transmembrane protein, has already been associated with many neurological disorders such as anxiety and panic disorders [61] and general behavioral disinhibition, including alcohol consumption and dependence, illicit drug use, and nicotine use [62]. There is no strong, widely replicated GWAS evidence that common variants in *TRAPPC8* and *PITPNM2* are a major, direct driver of cigarette quantity. Despite *TRAPPC8* is expressed in brain tissues and is a TRAPP complex subunit involved in vesicle trafficking/autophagy, no clear evidence in the literature highlights its possible role in smoking behaviour, despite future focus on rare variants could shed some light on it. On the other hand *PITPNM2* is involved in brain responses to drugs (changes in expression/DNA methylation and some exploratory signals), which makes it a plausible candidate in addiction-related biology [63]. A future step would be looking into possible pleiotropic effects of these genes with common actors in smoking behaviors.

## Conclusions

Polymorphic transposable elements emerge as a compelling avenue for elucidating human genetic diversity. The innovative use of polymorphic TEs as markers for genetic variability within isolated communities represents a promising methodological advancement. This study demonstrates the utility of polymorphic TEs in effectively encapsulating genetic variability and historical contexts among isolates, substantiated by congruent outcomes with prior investigations relying on single nucleotide variants [3–5]. While progress has been made, the comprehensive impact of transposable elements on the human genome remains incompletely understood, as does the cascade of effects on diverse phenotypes. This investigation identifies numerous TE insertions correlated with specific phenotypes, such as substance use and metabolic disorders. It is imperative to underscore the exploratory nature of our analyses, necessitating further empirical validation to establish definitive causal links between these insertions and medical susceptibility. Nevertheless, the identified insertions stand as pivotal points of interest, providing a foundational platform for subsequent research. Consequently, prospective studies should prioritize the validation of identified variants and engage in selection analyses to discern potential instances of natural selection within these isolated populations. This forward-looking research agenda holds significant promise for advancing our understanding of the intricate interplay between transposable elements and human phenotypic traits.

## Supplementary Information

Supplementary Material 1

## Data Availability

Genetic Data of isolated populations are available in the European Genome-phenome Archive (EGA) at the following links. BAM files: https://www.ebi.ac.uk/ega/studies/EGAS00001000252 (accessed on 21 st February 2024); sample list, vcf files: https://www.ebi.ac.uk/ega/studies/EGAS00001001597 (accessed on 21 st February 2024); https://www.ebi.ac.uk/ega/datasets/EGAD00001002729 (accessed on 21 st February 2024).

## References

[1] Sazzini M, Gnecchi Ruscone GA, Giuliani C, Sarno S, Quagliariello A, De Fanti S, et al. Complex interplay between neutral and adaptive evolution shaped differential genomic background and disease susceptibility along the Italian Peninsula. Sci Rep. 2016;6:32513. 27582244 10.1038/srep32513 PMC5007512

[2] Pesaresi S, Galdenzi D, Biondi E, Casavecchia S. Bioclimate of Italy: application of the worldwide bioclimatic classification system. J Maps. 2014;10(4):538–53. 10.1080/17445647.2014.891472.

[3] Esko T, Mezzavilla M, Nelis M, Borel C, Debniak T, Jakkula E, et al. Genetic characterization of Northeastern Italian population isolates in the context of broader European genetic diversity. Eur J Hum Genet. 2013;21:659–65. 23249956 10.1038/ejhg.2012.229 PMC3658181

[4] Cocca M, Barbieri C, Concas MP, Robino A, Brumat M, Gandin I, et al. A bird’s-eye view of Italian genomic variation through whole-genome sequencing. Eur J Hum Genet. 2020;28:435–44. 31784700 10.1038/s41431-019-0551-x PMC7080768

[5] Xue Y, Mezzavilla M, Haber M, McCarthy S, Chen Y, Narasimhan V, et al. Enrichment of low-frequency functional variants revealed by whole-genome sequencing of multiple isolated European populations. Nat Commun. 2017;8:15927. 28643794 10.1038/ncomms15927 PMC5490002

[6] Southam L, Gilly A, Süveges D, Farmaki A-E, Schwartzentruber J, Tachmazidou I, et al. Whole genome sequencing and imputation in isolated populations identify genetic associations with medically-relevant complex traits. Nat Commun. 2017;8:15606. 28548082 10.1038/ncomms15606 PMC5458552

[7] Rishishwar L, Tellez Villa CE, Jordan IK. Transposable element polymorphisms recapitulate human evolution. Mob DNA. 2015;6:21. 26579215 10.1186/s13100-015-0052-6 PMC4647816

[8] Gardner EJ, Lam VK, Harris DN, Chuang NT, Scott EC, Pittard WS, et al. The mobile element locator tool (MELT): population-scale mobile element discovery and biology. Genome Res. 2017;27:1916–29. 28855259 10.1101/gr.218032.116 PMC5668948

[9] Watkins WS, Feusier JE, Thomas J, Goubert C, Mallick S, Jorde LB. The Simons Genome Diversity Project: A Global Analysis of Mobile Element Diversity. Schaack S, editor. Genome Biology and Evolution [Internet]. 2020 [cited 2023 Oct 24];12:779–94. Available from: https://academic.oup.com/gbe/article/12/6/779/5828221 10.1093/gbe/evaa086 PMC7290288 32359137

[10] Perna NT, Batzer MA, Deininger PL, Stoneking M. Alu insertion polymorphism: a new type of marker for human population studies. Hum Biol. 1992;64:641–8. 1328024

[11] Batzer MA, Stoneking M, Alegria-Hartman M, Bazan H, Kass DH, Shaikh TH, et al. African origin of human-specific polymorphic Alu insertions. Proc Natl Acad Sci U S A. 1994;91:12288–92. 7991620 10.1073/pnas.91.25.12288 PMC45422

[12] Lee JY, Ji Z, Tian B. Phylogenetic analysis of mRNA polyadenylation sites reveals a role of transposable elements in evolution of the 3’-end of genes. Nucleic Acids Res. 2008;36:5581–90. 18757892 10.1093/nar/gkn540 PMC2553571

[13] Belancio VP, Roy-Engel AM, Deininger P. The impact of multiple splice sites in human L1 elements. Gene. 2008;411:38–45. 18261861 10.1016/j.gene.2007.12.022 PMC2278003

[14] Hata K, Sakaki Y. Identification of critical CpG sites for repression of L1 transcription by DNA methylation. Gene. 1997;189:227–34. 9168132 10.1016/s0378-1119(96)00856-6

[15] Enriquez-Gasca R, Gould PA, Rowe HM. Host gene regulation by transposable elements: the new, the old and the ugly. Viruses. 2020;12:1089. 32993145 10.3390/v12101089 PMC7650545

[16] Kim DS, Hahn Y. Identification of human-specific transcript variants induced by DNA insertions in the human genome. Bioinformatics. 2011;27:14–21. 21037245 10.1093/bioinformatics/btq612

[17] Pontis J, Planet E, Offner S, Turelli P, Duc J, Coudray A, et al. Hominoid-Specific transposable elements and KZFPs facilitate human embryonic genome activation and control transcription in Naive human ESCs. Cell Stem Cell. 2019;24:724–e7355. 31006620 10.1016/j.stem.2019.03.012 PMC6509360

[18] Cordaux R, Batzer MA. The impact of retrotransposons on human genome evolution. Nat Rev Genet. 2009;10:691–703. 19763152 10.1038/nrg2640 PMC2884099

[19] Anwar SL, Wulaningsih W, Lehmann U. Transposable elements in human cancer: causes and consequences of deregulation. Int J Mol Sci. 2017;18:974. 28471386 10.3390/ijms18050974 PMC5454887

[20] Chénais B. Transposable elements and human diseases: mechanisms and implication in the response to environmental pollutants. Int J Mol Sci. 2022;23:2551. 35269693 10.3390/ijms23052551 PMC8910135

[21] Kazazian HH, Wong C, Youssoufian H, Scott AF, Phillips DG, Antonarakis SE. Haemophilia a resulting from de novo insertion of L1 sequences represents a novel mechanism for mutation in man. Nature. 1988;332:164–6. 2831458 10.1038/332164a0

[22] Nakamura Y, Murata M, Takagi Y, Kozuka T, Nakata Y, Hasebe R, et al. SVA retrotransposition in exon 6 of the coagulation factor IX gene causing severe hemophilia B. Int J Hematol. 2015;102:134–9. 25739383 10.1007/s12185-015-1765-5

[23] Payer LM, Burns KH. Transposable elements in human genetic disease. Nat Rev Genet. 2019;20:760–72. 31515540 10.1038/s41576-019-0165-8

[24] Jelassi A, Slimani A, Rabès JP, Jguirim I, Abifadel M, Boileau C, et al. Genomic characterization of two deletions in the LDLR gene in Tunisian patients with familial hypercholesterolemia. Clin Chim Acta. 2012;414:146–51. 22910581 10.1016/j.cca.2012.08.002

[25] Reilly MT, Faulkner GJ, Dubnau J, Ponomarev I, Gage FH. The role of transposable elements in health and diseases of the central nervous system. J Neurosci. 2013;33:17577–86. 24198348 10.1523/JNEUROSCI.3369-13.2013 PMC3818539

[26] Manolio TA, Collins FS, Cox NJ, Goldstein DB, Hindorff LA, Hunter DJ, et al. Finding the missing heritability of complex diseases. Nature. 2009;461:747–53. 19812666 10.1038/nature08494 PMC2831613

[27] Erwin JA, Marchetto MC, Gage FH. Mobile DNA elements in the generation of diversity and complexity in the brain. Nat Rev Neurosci. 2014;15:497–506. 25005482 10.1038/nrn3730 PMC4443810

[28] Rau V, Fanselow MS. Exposure to a stressor produces a long lasting enhancement of fear learning in rats. Stress. 2009;12:125–33. 18609302 10.1080/10253890802137320

[29] Ponomarev I, Rau V, Eger EI, Harris RA, Fanselow MS. Amygdala transcriptome and cellular mechanisms underlying stress-enhanced fear learning in a rat model of posttraumatic stress disorder. Neuropsychopharmacology. 2010;35:1402–11. 20147889 10.1038/npp.2010.10 PMC3040562

[30] Grucza RA, Bierut LJ. Cigarette smoking and the risk for alcohol use disorders among adolescent drinkers. Alcohol Clin Exp Res. 2006;30:2046–54. 17117970 10.1111/j.1530-0277.2006.00255.x PMC2431150

[31] Rehm J. The risks associated with alcohol use and alcoholism. Alcohol Res Health. 2011;34:135–43. 22330211 PMC3307043

[32] Le Foll B, Piper ME, Fowler CD, Tonstad S, Bierut L, Lu L et al. Tobacco and nicotine use. Nat Rev Dis Primers [Internet]. 2022 [cited 2025 Sept 14];8:19. Available from: https://www.nature.com/articles/s41572-022-00346-w 10.1038/s41572-022-00346-w 35332148

[33] Li H, Durbin R. Fast and accurate short read alignment with Burrows-Wheeler transform. Bioinformatics. 2009;25:1754–60. 19451168 10.1093/bioinformatics/btp324 PMC2705234

[34] Sherman BT, Hao M, Qiu J, Jiao X, Baseler MW, Lane HC, et al. DAVID: a web server for functional enrichment analysis and functional annotation of gene lists (2021 update). Nucleic Acids Res. 2022;50:W216–21. 35325185 10.1093/nar/gkac194 PMC9252805

[35] 1000 Genomes Project Consortium, Auton A, Brooks LD, Durbin RM, Garrison EP, Kang HM, et al. A global reference for human genetic variation. Nature. 2015;526:68–74. 26432245 10.1038/nature15393 PMC4750478

[36] Purcell S, Neale B, Todd-Brown K, Thomas L, Ferreira MAR, Bender D et al. PLINK: A Tool Set for Whole-Genome Association and Population-Based Linkage Analyses. The American Journal of Human Genetics [Internet]. 2007 [cited 2023 Oct 24];81:559–75. Available from: https://linkinghub.elsevier.com/retrieve/pii/S0002929707613524 10.1086/519795 PMC1950838 17701901

[37] Chen B, Cole JW, Grond-Ginsbach C. Departure from hardy Weinberg equilibrium and genotyping error. Front Genet. 2017;8:167. 29163635 10.3389/fgene.2017.00167 PMC5671567

[38] Price AL, Patterson NJ, Plenge RM, Weinblatt ME, Shadick NA, Reich D. Principal components analysis corrects for stratification in genome-wide association studies. Nat Genet. 2006;38:904–9. 16862161 10.1038/ng1847

[39] Alexander DH, Lange K. Enhancements to the ADMIXTURE algorithm for individual ancestry estimation. BMC Bioinformatics. 2011;12:246. 21682921 10.1186/1471-2105-12-246 PMC3146885

[40] Pickrell JK, Pritchard JK. Inference of Population Splits and Mixtures from Genome-Wide Allele Frequency Data. Tang H, editor. PLoS Genet [Internet]. 2012 [cited 2025 Sept 14];8:e1002967. Available from: 10.1371/journal.pgen.1002967 10.1371/journal.pgen.1002967 PMC3499260 23166502

[41] Lek M, Karczewski KJ, Minikel EV, Samocha KE, Banks E, Fennell T, et al. Analysis of protein-coding genetic variation in 60,706 humans. Nature. 2016;536:285–91. 27535533 10.1038/nature19057 PMC5018207

[42] Petrovski S, Wang Q, Heinzen EL, Allen AS, Goldstein DB. Genic intolerance to functional variation and the interpretation of personal genomes. PLoS Genet. 2013;9:e1003709. 23990802 10.1371/journal.pgen.1003709 PMC3749936

[43] Mezzavilla M, Cocca M, Guidolin F, Gasparini P. A population-based approach for gene prioritization in understanding complex traits. Hum Genet. 2020;139:647–55. 32232557 10.1007/s00439-020-02152-4

[44] Zhou X, Stephens M. Genome-wide efficient mixed-model analysis for association studies. Nat Genet. 2012;44:821–4. 22706312 10.1038/ng.2310 PMC3386377

[45] Zhou X, Stephens M. Efficient multivariate linear mixed model algorithms for genome-wide association studies. Nat Methods. 2014;11:407–9. 24531419 10.1038/nmeth.2848 PMC4211878

[46] Browning SR, Browning BL. Rapid and accurate haplotype phasing and missing-data inference for whole-genome association studies by use of localized haplotype clustering. Am J Hum Genet. 2007;81:1084–97. 17924348 10.1086/521987 PMC2265661

[47] Danecek P, Auton A, Abecasis G, Albers CA, Banks E, DePristo MA, et al. The variant call format and vcftools. Bioinformatics. 2011;27:2156–8. 21653522 10.1093/bioinformatics/btr330 PMC3137218

[48] Cao X, Zhang Y, Payer LM, Lords H, Steranka JP, Burns KH, et al. Polymorphic mobile element insertions contribute to gene expression and alternative splicing in human tissues. Genome Biol. 2020;21(1):185. 10.1186/s13059-020-02101-4. 32718348 10.1186/s13059-020-02101-4 PMC7385971

[49] Wang H, Xing J, Grover D, Hedges DJ, Han K, Walker JA, et al. SVA elements: a hominid-specific retroposon family. J Mol Biol. 2005;354:994–1007. 16288912 10.1016/j.jmb.2005.09.085

[50] Gianfrancesco O, Bubb VJ, Quinn JP. SVA retrotransposons as potential modulators of neuropeptide gene expression. Neuropeptides. 2017;64:3–7. 27743609 10.1016/j.npep.2016.09.006 PMC5529292

[51] Barnada SM, Isopi A, Tejada-Martinez D, Goubert C, Patoori S, Pagliaroli L, et al. Genomic features underlie the co-option of SVA transposons as cis-regulatory elements in human pluripotent stem cells. PLoS Genet. 2022;18:e1010225. 10.1371/journal.pgen.1010225. 35704668 10.1371/journal.pgen.1010225 PMC9239442

[52] Charlesworth B. Fundamental concepts in genetics: effective population size and patterns of molecular evolution and variation. Nat Rev Genet. 2009;10:195–205. 19204717 10.1038/nrg2526

[53] Hatzikotoulas K, Gilly A, Zeggini E. Using population isolates in genetic association studies. Brief Funct Genomics. 2014;13:371–7. 25009120 10.1093/bfgp/elu022 PMC4168662

[54] Destro Bisol G, Anagnostou P, Batini C, Battaggia C, Bertoncini S, Boattini A, et al. Italian isolates today: geographic and linguistic factors shaping human biodiversity. J Anthropol Sci. 2008;86:179–88. 19934475

[55] Platzer K, Lemke JR. GRIN2B-Related Neurodevelopmental Disorder. In: Adam MP, Feldman J, Mirzaa GM, Pagon RA, Wallace SE, Bean LJ, editors. GeneReviews^®^ [Internet]. Seattle (WA): University of Washington, Seattle; 1993 [cited 2024 Jan 29]. Available from: http://www.ncbi.nlm.nih.gov/books/NBK501979/29851452

[56] Saunders GRB, Wang X, Chen F, Jang S-K, Liu M, Wang C et al. Genetic diversity fuels gene discovery for tobacco and alcohol use. Nature [Internet]. 2022 [cited 2024 Jan 29];612:720–4. Available from: https://www.nature.com/articles/s41586-022-05477-4 10.1038/s41586-022-05477-4 PMC9771818 36477530

[57] Karlsson Linnér R, Biroli P, Kong E, Meddens SFW, Wedow R, Fontana MA, et al. Genome-wide association analyses of risk tolerance and risky behaviors in over 1 million individuals identify hundreds of loci and shared genetic influences. Nat Genet. 2019;51:245–57. 30643258 10.1038/s41588-018-0309-3 PMC6713272

[58] Sherva R, Zhu C, Wetherill L, Edenberg HJ, Johnson E, Degenhardt L, et al. Genome-wide association study of phenotypes measuring progression from first cocaine or opioid use to dependence reveals novel risk genes. Explor Med. 2021;2:60–73. 34124712 10.37349/emed.2021.00032 PMC8192073

[59] Goes FS, McGrath J, Avramopoulos D, Wolyniec P, Pirooznia M, Ruczinski I, et al. Genome-wide association study of schizophrenia in Ashkenazi Jews. Am J Med Genet B Neuropsychiatr Genet. 2015;168:649–59. 26198764 10.1002/ajmg.b.32349

[60] Kulminski AM, Loiko E, Loika Y, Culminskaya I. Pleiotropic predisposition to Alzheimer’s disease and educational attainment: insights from the summary statistics analysis. Geroscience. 2022;44:265–80. 34743297 10.1007/s11357-021-00484-1 PMC8572080

[61] Otowa T, Maher BS, Aggen SH, McClay JL, van den Oord EJ, Hettema JM. Genome-wide and gene-based association studies of anxiety disorders in European and African American samples. PLoS ONE. 2014;9:e112559. 25390645 10.1371/journal.pone.0112559 PMC4229211

[62] McGue M, Zhang Y, Miller MB, Basu S, Vrieze S, Hicks B, et al. A genome-wide association study of behavioral disinhibition. Behav Genet. 2013;43:363–73. 23942779 10.1007/s10519-013-9606-x PMC3886341

[63] Massart R, Barnea R, Dikshtein Y, Suderman M, Meir O, Hallett M, et al. Role of DNA methylation in the nucleus accumbens in incubation of cocaine craving. J Neurosci. 2015;35:8042–58. 26019323 10.1523/JNEUROSCI.3053-14.2015 PMC6605346

