# Supplementary material for: Variability of transposable elements in six genetic isolates from North-Eastern Italy and their relationship with alcohol consumption, tobacco use and BMI
Source: BMC Genomics. 2025 Nov 11;26:1027. doi: 10.1186/s12864-025-12225-1 (PMC12607106; doi:10.1186/s12864-025-12225-1)
Supplement: Supplementary file 1 — Supplementary Material 1 [file 12864_2025_12225_MOESM1_ESM.pdf]

Variability of Transposable Elements in six genetic isolates from North-Eastern Italy and their relationship with alcohol consumption, tobacco use and BMI

Supplementary Materials

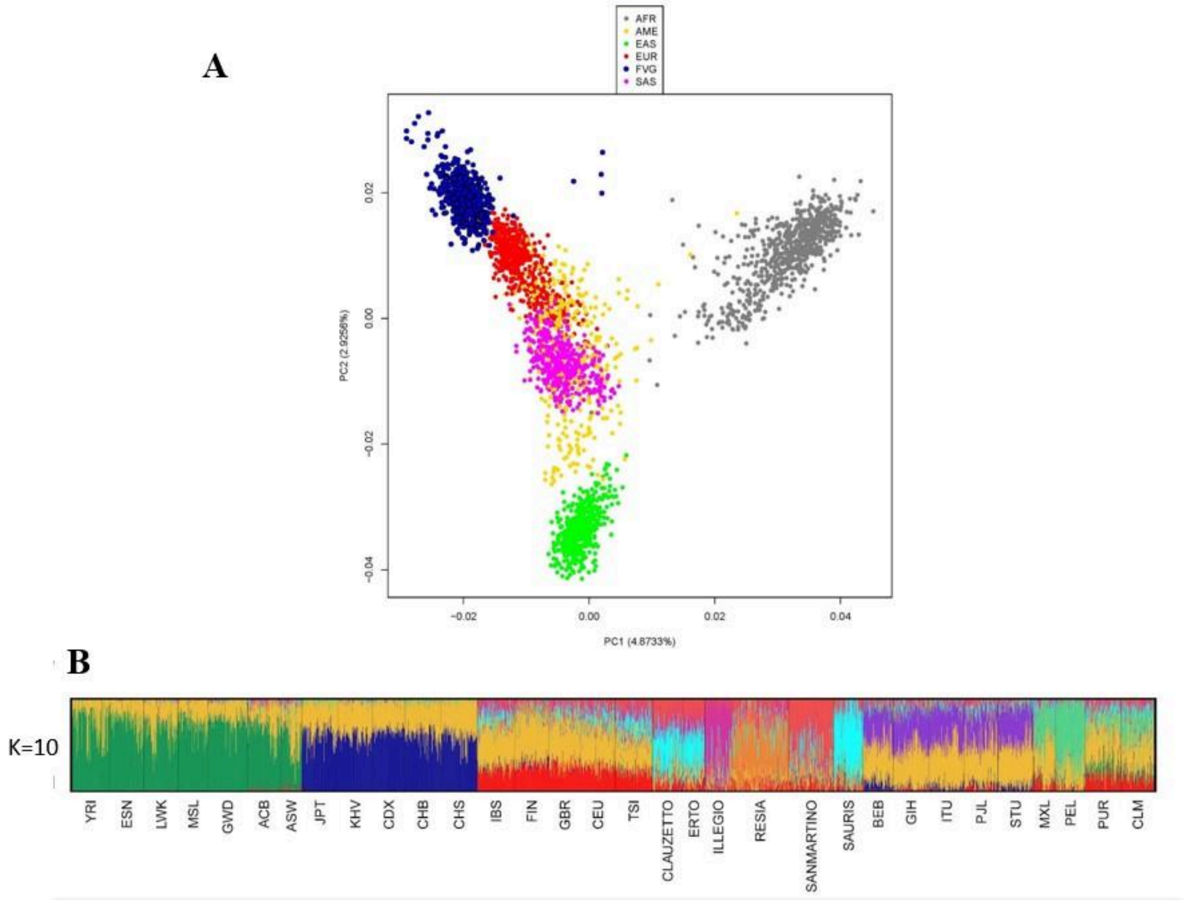

Supplementary Figure S1. A) Worldwide PCA plot including FVG isolates and all the 26 populations of the 1000 Genomes Project, divided by macro-areas: AFR=Africa; AME=America; EAS=East Asia; FVG=Friuli Venezia-Giulia; SAS=South Asia. B) Admixture plot at best fitting K=10 with all the 26 populations of the 1000 Genomes Project, plus the six isolates of FVG. IDs of the 26 1KGP populations are the same used by the project.

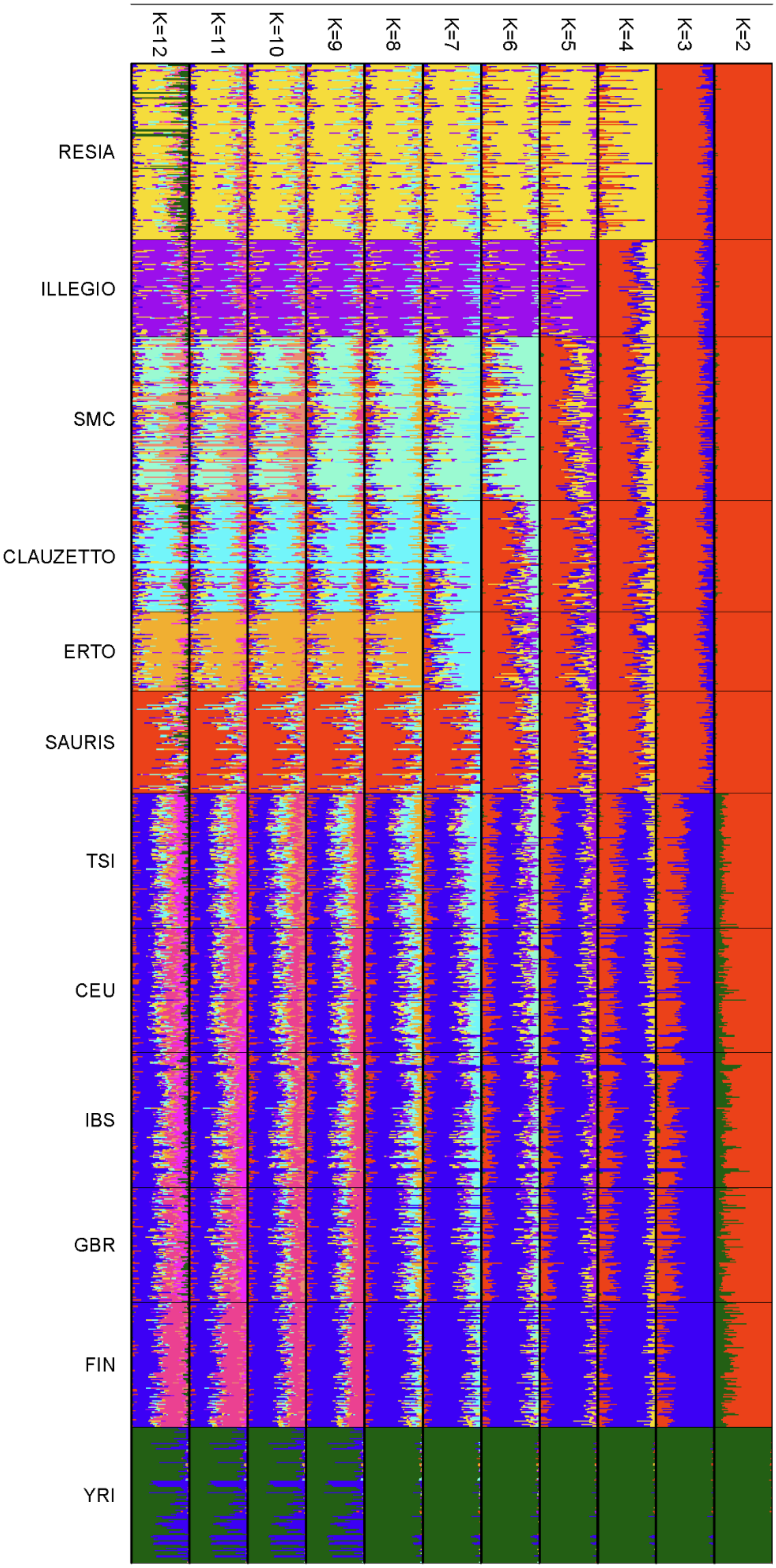
K=12
K=11
K=10
K=9
K=8
K=7
K=6
K=5
K=4
K=3
K=2
RESIA
ILLEGIO
SMC
CLAUZETTO
ERTO
SAURIS
TSI
CEU
IBS
GBR
FIN
YRI

Supplementary Figure S2 (continues from the previous page). Admixture plots for all tested Ks (0-12) with the 7 isolated villages, European populations and YRI as an outgroup. IDs of the 1KGP populations are the same as the ones used in the project.

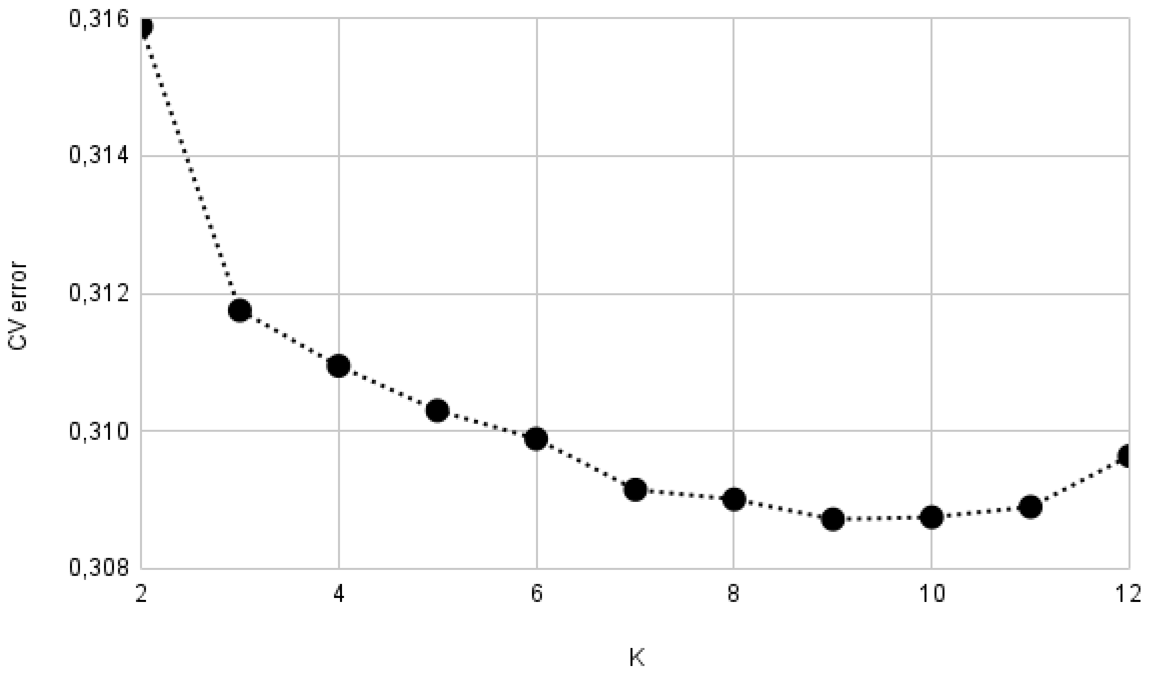

Supplementary Figure S3. Scatterplot graph highlighting cross-validation (CV) errors for each tested ancestry (K) for the 12 ADMIXTURE runs including the 7 villages, European populations and the African YRI as an outgroup.

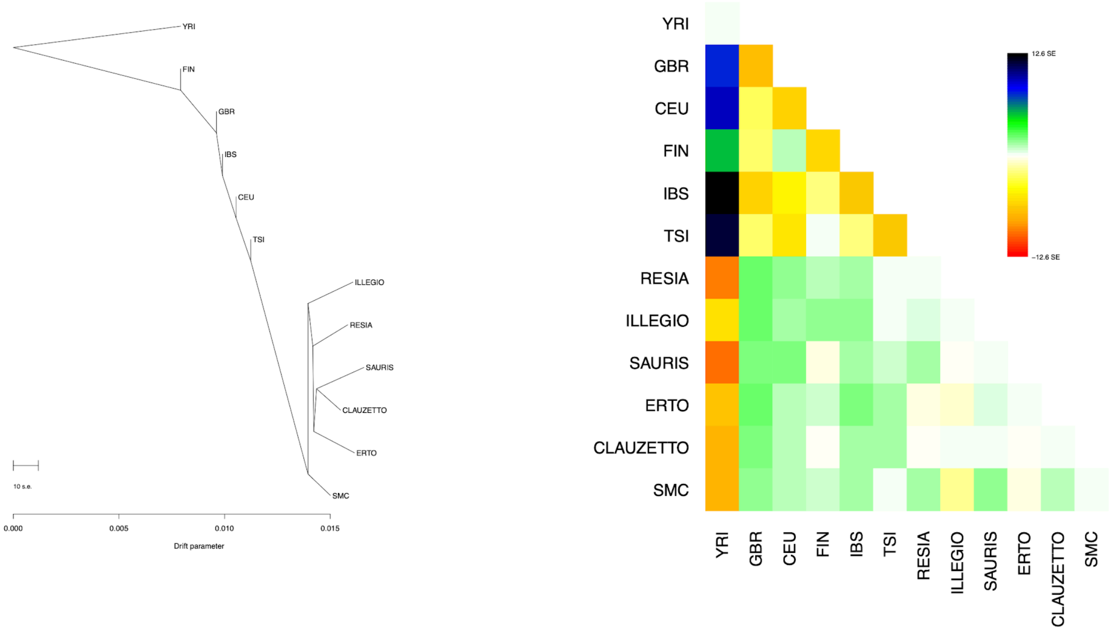

<u>Supplementary Figure S4.</u> TreeMix analyses. On the left the relationship graph is shown, which shows how the isolated populations cluster together after splitting from the TSI population. On the right, the residual variation of TreeMix analyses, a high positive value indicates possible evidence of additional gene flow between populations, and it shows how the isolated populations have no evidence of gene flow from the reference populations.

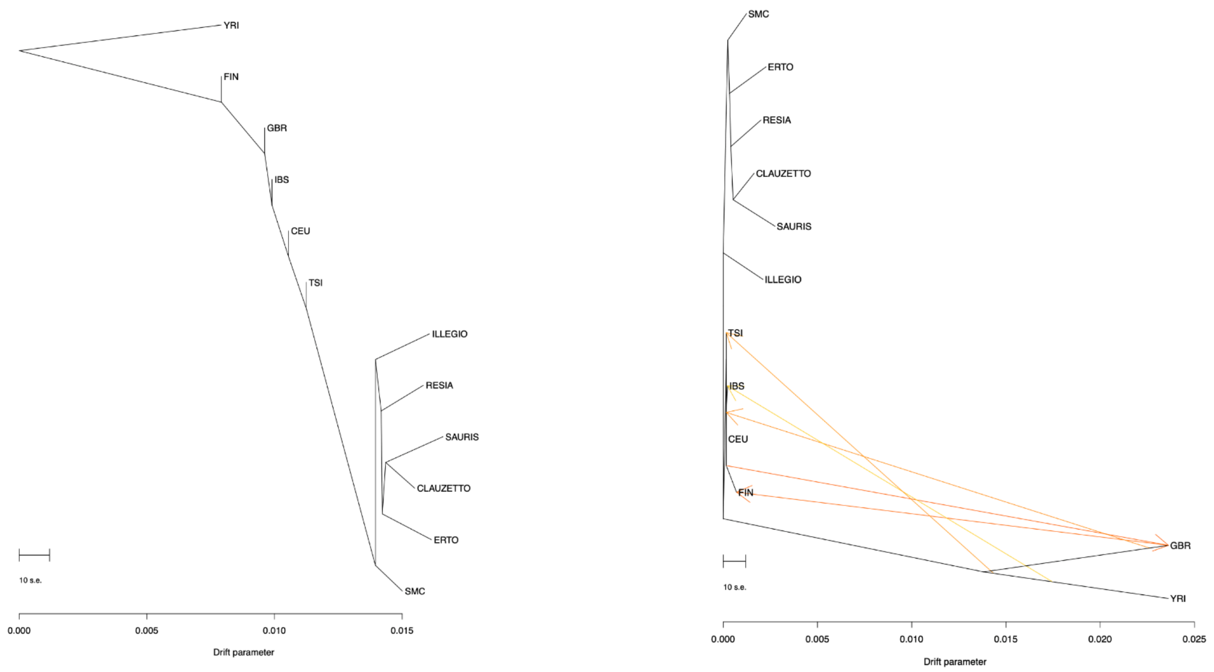

Supplementary Figure S5. TreeMix analyses with migration edges. On the left, tree with 0 migration edges. On the right, tree with 5 migration edges, explaining 99.9% of the variance.

Position/Hg38	TE Type	Gene ID	Insertion location
chr1:100366239	Alu	AGL	EXON 24
chr1:109648713	Alu	CFAP276	EXON 5
chr1:65272991	Alu	RAVER2	EXON 9
chr2:170092544	Alu	LRP2	EXON 29
chr2:202590094	Alu	ALS2	EXON 20
chr2:220073207	Alu	ZFAND2B	EXON 6
chr2:73496597	Alu	FBXO41	EXON 1
chr2:84931310	Alu	DNAH6	EXON 51
chr3:113015598	Alu	CFAP44	EXON 33
chr4:128949763	LINE1	ABHD18	EXON 10
chr4:30921811	Alu	PCDH7	EXON 2
chr5:140215553	Alu	PCDHA7	EXON 1
chr5:149677538	Alu	ARSI	EXON 2
chr5:176306406	SVA	UNC5A	EXON 12
chr6:101215134	Alu	ASCC3	EXON 9
chr6:129786303	LINE1	LAMA2	EXON 51
chr6:155116248	Alu	SCAF8	EXON 6
chr6:32805660	Alu	TAP2	EXON 2
chr6:4954206	Alu	CDYL	EXON 7
chr7:107848084	SVA	NRCAM	EXON 12
chr7:127230174	Alu	ARF5	EXON 4

Position/Hg38	TE Type	Gene ID	Insertion location
chr7:128852188	Alu	SMO	EXON 12
chr7:140476723	Alu	BRAF	EXON 13
chr8:10468773	Alu	RP1L1	EXON 4
chr9:100616862	LINE1	FOXE1	EXON 1
chr10:93242752	Alu	HECTD2	EXON 8
chr12:121881827	Alu	KDM2B	EXON 16
chr12:55714528	Alu	OR6C1	EXON 1
chr13:35751205	SVA	NBEA	EXON 12
chr14:24523719	LINE1	CARMIL3	EXON 5
chr14:34269852	Alu	NPAS3	EXON 12
chr15:23811635	Alu	MKRN3	EXON 1
chr15:25924678	Alu	ATP10A	EXON 21
chr15:50897115	LINE1	TRPM7	EXON 21
chr16:2052233	Alu	ZNF598	EXON 7
chr16:2374443	SVA	ABCA3	EXON 12
chr16:85141466	Alu	CIBAR2	EXON 4
chr17:16630902	SVA	CCDC144A	EXON 12
chr17:56065448	LINE1	VEZF1	EXON 1
chr17:71232302	Alu	C17orf80	EXON 3
chr18:34647028	Alu	KIAA1328	EXON 7
chr19:11727948	Alu	ZNF627	EXON 4
chr19:32954844	Alu	DPY19L3	EXON 14

Position/Hg38	TE Type	Gene ID	Insertion location
chr19:36222848	SVA	KMT2B	EXON 12
chr19:46443685	Alu	NOVA2	EXON 4
chr19:57869033	Alu	ZNF304	EXON 3
chr19:9721262	Alu	ZNF561	EXON 6
chr20:30345355	Alu	TPX2	EXON 3
chr20:3180667	Alu	DDRGK1	EXON 4
chr20:35129001	Alu	DLGAP4	EXON 4

Supplementary Table S1 (continues from the previous pages). List of polymorphic TEs within exons. Positions (in human reference genome hg38), TE type and gene ID are shown.

K	CV Error	LogLikelihood
2	0,31588	-1122673,235654
3	0,31175	-1111262,937266
4	0,31095	-1105808,464568
5	0,31030	-1101194,454838
6	0,30989	-1096996,852554
7	0,30915	-1092951,724377
8	0,30901	-1089459,898436
9	0,30872	-1086493,212541
10	0,30875	-1083840,100963
11	0,30890	-1081309,344333
12	0,30964	-1078983,912422

Supplementary Table S2. List of cross validation (CV) errors and LogLikelihood values associated with each tested ancestry number (K) for the ADMIXTURE run including the 7 isolated villages, European populations, and African YRI as an outgroup.

chr:position	chr17:49150166	chr12:14020945	chr12:129970510	chr12:123580101	chr18:29519986
**TE type**	SVA	Alu	Alu	Alu	Alu
**Gene**	**SPAG9**	**GRIN2B**	**TMEM132D**	**PITPNM2**	**TRAPPC8**
**Association test**	BMI_sex_age	Alcohol_sex_age	Smoke_sex_age	N_cigarettes_N_years	N_cigarettes_N_years
**beta_1**	-0,0155	0,0652	-0,106	-2,06	-3,4
**beta_2**	-6,59	-2,28	1,89	35,8	43,4
**beta_3**	2,61	0,105	0,0602		
**Vbeta_1_1**	0,013	0,000974	0,00112	36,6	36,4
**Vbeta_1_2**	-0,0187	-0,00113	-0,000807	6,7	7,51
**Vbeta_1_3**	0,0252	0,000202	-0,0000102		
**Vbeta_2_2**	13,4	1,11	1,15	70,7	64,4
**Vbeta_2_3**	1,47	0,00136	-0,0022		
**Vbeta_3_3**	0,998	0,000918	0,000773		
**SE_beta1**	0,114017543	0,031208973	0,033466401	6,049793385	6,033241252
**SE_beta2**	3,660601044	1,053565375	1,072380529	8,408329204	8,024961059
**SE_beta3**	0,998999499	0,030298515	0,027802878		
**p score**	0,000442	0,000268	0,000456	0,000177	0,0000013

Supplementary Table S3. GEMMA output for constrained genes showing significant results. For each significant insertion, effect sizes, associated standard error and p-values are reported.
